# Social Aspects of Dementia Prevention from a Worldwide to National Perspective: A Review on the International Situation and the Example of Italy

**DOI:** 10.1155/2019/8720904

**Published:** 2019-09-08

**Authors:** Giovanna Ricci

**Affiliations:** School of Law, University of Camerino, Camerino, Italy

## Abstract

At the moment, dementia is affecting around 47 million people worldwide, with a forecast amount of 135 million affected people in 2050. Dementia is a growing health concern worldwide with no treatment currently available, but only symptomatic medication. Effective interventions in the prevention and management of dementia are urgently needed to contain direct and indirect costs of this disease. Indeed, the economic impact of dementia is a vast and continually growing figure, but it is still difficult to quantify. Due to an increase in both the disease spreading and its direct and indirect costs, national and international action plans have to be implemented. As a virtuous example, the Italian national plan for dementia has been summarized. Faced with an increasingly less sustainable disease impact at national and international levels, the plan suggests that it is certainly the entire welfare model that should be rethought, strengthening the network of services and providing interventions to support affected people and their caregivers. Alongside this synergistic approach, scientific research could play a crucial role for pharmacological and nonpharmacological treatments capable of delaying the state of loss of self-sufficiency of the patient, with a significant impact on social and health costs.

## 1. Introduction

More than 47 million people are affected worldwide by dementia, and the majority of them are over 65 years old. For this reason, this phenomenon is considered a growing health concern, given the increased longevity of the world population (especially in developed countries) combined with the absence of a treatment capable of modifying the disease [1]. This occurrence creates an urgent need for effective interventions with respect to prevention and disease management.

Priorities on the dementia phenomena are comprehension of the pathology in terms of cellular, molecular, and genetic processes and early diagnosis through the use of cognitive tests and clinical trials, but mainly the understanding of social aspects including social programs and technology to benefit medical care and programs to contain the costs of the disease.

The most common forms of dementia are the vascular and the Alzheimer variations, differentiable by measuring specific biomarkers in biological fluids, particularly in the cerebrospinal fluid (CSF), and by imaging these biomarkers. Both approaches are of extreme importance in optimizing a precise early clinical diagnosis and predicting the outcome in particular settings [2, 3].

The chronically degenerative process inducted by dementia includes a set of conditions such as functional and behavioural alterations, dynamic progression of cognitive disabilities, loss of self-sufficiency, and increasing dependence on caregivers. In this sense, the disease starts interfering with working abilities and social interactions and ends with more or less total dependency on others, and the damage done by the disease is irreversible.

Mild cognitive impairment is considered the early stage of any form of dementia and is characterized by a light cognitive decrease in comparison to a previous level of capability. This decrease poorly interferes with the normal activities of the patient during the daily life, and this condition is mainly identified in specialized centres. This impairment is present in about 19% of people over 65. The conversion rate in dementia is 46% in three years [4]. Considering that many patients do not have a direct diagnosis of dementia at its early stages, it can be stated that the former data underestimates the problem. At the same time, it cannot be ignored that there are also rates of reversion to normal cognition varying from 29 to 55% in population-based cohort studies and from 4 to 15% in clinical settings [5].

The two screening tools currently used for diagnosis are the General Practitioner Assessment of Cognition (GPCog) and the clock-drawing test, both used to screen cognitive impairment and dementia and as measures of spatial dysfunction and neglect. The GPCog consists of a four-component patient assessment and a brief informant interview (six questions) [6], whereas in the clock-drawing test, the subject is simply asked to draw a clock from memory [7]. Besides these two principal methods to screen dementia, there are several other tools including the Montreal Cognitive Assessment, the Mini-Mental State Examination, and memory- and executive function-specific measures [8, 9].

Patients with signs of cognitive decline could then be referred to specialized structures, to undergo deeper investigation.

In reversible forms of dementia, whose prevalence is highly variable (8-40%, with an approximate general value of 12% in patients presenting themselves at services with symptoms) depending on the clinical evaluation and the sociodemographic features of patients [10], the deficits are secondary and, if timely and appropriately cared for, the deterioration can regress and the patient returns to their standard level of capability [2].

Irreversible dementias can be distinguished into a primary and a secondary form. In the primary form, the disease is degenerative and includes Alzheimer's disease, frontotemporal dementia, and dementia with Lewy bodies. In secondary dementia, the vascular variation is predominant [2].

At initial and intermediate stages, symptomatic characteristics of all forms of irreversible dementia are quite distinguishable from each other; this difference in fact decreases and disappears completely with the progress of the degeneration [2].

“Major neurocognitive disorders” are the current formal identification of dementia, according to the *Diagnostic and Statistical Manual of Mental Disorders* (DSM) 5 [11], characterizing them as illnesses where the neural substrate anomaly is demonstrable, together with a cognitive deficiency, in patients with previous normal brain capability [12].

More specifically, in 2011, the National Institute on Aging and the Alzheimer's Association (NIA/AA) in the United States published new diagnostic guidelines for Alzheimer's disease focusing on three stages of the disorder: dementia due to Alzheimer's disease, mild cognitive impairment (MCI) due to Alzheimer's disease, and preclinical (presymptomatic) Alzheimer's disease [13–15].

In dementia due to Alzheimer's disease, impairments in memory, thinking, and behaviour reduce the ability to autonomously operate in everyday life. In mild cognitive impairment due to Alzheimer's disease, mild changes in memory and thinking are noticeable and measurable with mental status tests but are not severe enough to disrupt a person's day-to-day life. Differently, preclinical Alzheimer's disease is a newly defined stage of the disease where measureable biomarker changes in the brain may occur years before symptoms affecting memory, thinking, or behaviour can be detected. In all these three stages, additional research is advocated to explore if biomarkers exist to detect each of the stages and “which biomarkers may best confirm that Alzheimer's-related changes are underway and how best to measure them” [13–15].

### 1.1. Epidemiology

Dementia hits an increasingly high number of people: in 2015, about 47 million people were affected and in 2050, 135 million patients are expected, with a prevalence of about 8% people over 65 years old in industrialized countries, rising to more than 20% in eight years. Due to these numbers, the World Health Organization (WHO) and Alzheimer's Disease International (ADI) have defined dementia as a world health priority [1].

According to the World Alzheimer Report [1], in 2015, the world regional distribution of new dementia cases was 4.9 million (49% of the total) in Asia, 2.5 million (25%) in Europe, 1.7 million (18%) in the Americas, and 0.8 million (8%) in Africa. Compared to the previous 2012 estimates, these values represent an increased proportion of new cases arising in Asia, the Americas, and Africa, while the proportion arising in Europe has dropped [12].

The evolution of dementia epidemiology is nonhomogeneous: according to international studies [16–18], countries like Sweden, Canada, the UK, the Netherlands, and the USA experienced a decrease in dementia prevalence or incidence, with a stable situation in Nigeria [19] and an increase in incidence in China [20] and in prevalence in Japan [21]. Despite the general increase in the absolute population, two recent studies showed a decrease in prevalence for age-specific segments [18, 22].

Over the next 20 years, an important increase in cases of dementia is expected in China, India, and sub-Saharan Africa [23]. Italy ranks second for the world's oldest population behind Japan and before Germany and Portugal: 21.4% people are over 65 and 6.4% are over 80 [1]. In the next 20 years or so, Chile, China, Iran, and Russia are expected to have a proportion of elderly population similar to Japan. By 2050, most people over 60 years old—120 million—will live in China, while 434 million will live in the rest of the world.

### 1.2. Dementia in Sub-Saharan Africa and Other Developing Countries: Increasing Evidence Highlighting the Current and Future Burden

About 4 million people with dementia were living in Africa in 2015, with over 27 million people living in other developing countries [1].

In 1990, Nigeria started the investigation on dementia prevalence in sub-Saharan countries, with an epidemiological study (Ibadan-Indianapolis Dementia Project). In that concern, community-based researches were indicating that the disease (Alzheimer dementia) was uncommon. After twenty years, sub-Saharan Africa is still poorly covered by studies (only nine studies identified the diseases thus far, carried in Tanzania, Benin, Central African Republic, Nigeria, and the Republic of Congo), but there is improving evidence of dementia prevalence.

According to the World Alzheimer Report 2015 [1], in this region of the world, there may be a 63% increase by 2030 in the population living with dementia, with a further increase of 257% by 2050.

Conversely, no information is currently available concerning any variation in the epidemiology of dementia in EU countries and particularly in Italy due to recent immigration flows from Africa. Indeed, it has to be considered that immigration from Africa is still a rising phenomenon involving very young people, so that it is not possible to predict the disease's prevalence in EU.

### 1.3. Effects of Potentially Modifiable Risk Factors on the Brain

There is a combination of nine risk factors for dementia in about 35% cases: education limited to the age of 12, midlife obesity and hypertension, late-life depression, loss of hearing, smoking, diabetes, poor physical activity, and social isolation [24].

On the other hand, it has been demonstrated that the complete elimination of the apolipoprotein E (ApoE) *ε*4 allele, which represents the major genetic risk, can lead to a 7% decrease in disease incidence [25].

According to the current international studies, it is observed that several dementia manifestations can now be managed, and even if the illness per se is not curable, the evolution may be modified with proper dementia care.

The following is a list of modifiable risk factors for dementia:

- Vascular brain damage, which increases the risk of macrovascular and microvascular lesions, atrophy, and neurodegeneration [26]
- Inflammation and oxidative stress, which are associated with deposition of amyloid beta peptide [27]
- Metabolic syndrome and diabetes, which are associated with pathologies like atherosclerosis or brain infarction and glucose-mediated toxicity responsible for microvascular abnormalities and neurodegeneration [28]

There is also evidence of impaired insulin receptor activation in Alzheimer's disease [29], suggesting that it might represent a brain state that is insulin-resistant [30].

### 1.4. Pharmacological Treatments in Dementias

Starting from 1998, only four of the 100 drugs tested against dementia have been authorized for use worldwide. In few cases, they can help manage several symptoms, but most people cannot access them. In addition, although many dementia manifestations are now manageable, the underlying illness is generally not curable, even if modifiable with good dementia care. Currently, even if a number of studies are trying to find effective treatment for dementias, only symptomatic treatments are available in practice. Two types of drugs are mainly accessible: The cholinesterase inhibitors—galantamine, rivastigmine, and above all donepezil [31]—which prevent the acetylcholinesterase enzyme from breaking down acetylcholine, and the neurotransmitter that connects nerve cells and keeps memory functioning. The other type of drug is memantine, an NMDA receptor antagonist that improves memory by restoring homeostasis in the glutamatergic system. Memantine blocks the effects of glutamate, which is released in excessive amounts in the brain of people affected by Alzheimer's disease.

Since it has been shown that Alzheimer's disease causes an imbalance between overproduction and inadequate clearance of the amyloid beta peptide that accumulates in the brain in plaques, drugs have been developed with the aim of stopping this phenomenon. One of these drugs is crenezumab consisting of monoclonal antibodies targeting beta-amyloid, and the other is gantenerumab, a human IgG1 antibody centrally acting to disassemble and degrade amyloid plaques [32].

In addition, a cholesterol-controlling drug, gemfibrozil, has been found to reduce amyloid levels and brain inflammation, at least in an animal model in mice and another humanized IgG1 version of the mouse monoclonal antibody mAb158, and BAN2401 has been found to reduce the amyloid beta peptide in the brain of 81% patients and slow cognitive decline in the brain by 30% [33].

In Italy, the only approved drugs to treat Alzheimer dementia are memantine and reversible acetylcholinesterase inhibitors: donepezil, rivastigmine, and galantamine.

These treatments are all regulated and prescribed by the Italian Drug Agency (AIFA) in charge of drug regulation and prescription [34]. To treat typical and atypical behavioural disorders in dementia, AIFA recommends the use of antipsychotic drugs.

### 1.5. Nonpharmacological Treatments

For the prevention of dementia or as support for other treatments, nonpharmacological treatments are crucial. There are two classes of prevention strategies: lifestyle-associated and diet-associated interventions.

#### 1.5.1. Lifestyle Strategies

Lifestyle strategies include caloric restriction, cognitive stimulation, socialization, and physical activity [35].

According to several studies [36, 37], physical activity is associated with the reduction of Alzheimer dementia prevalence [38]. Physical activity is associated with an improvement of hippocampal neurogenesis [38] and learning capability, as demonstrated in rodents [39].

Two explanations of the neuroprotective effect of physical activity were proposed:

1. Release of various neurotrophic factors (e.g., BDNF, IGF-1, NGF, and VEGF), stimulating the transcription factor (CREB) and activating the neurogenesis and the synaptic neuronal plasticity [39]
2. Reduction of free radicals in the hippocampus and a concurrent increase in superoxide dismutase and nitric oxide endothelial synthase [40]

Although there are studies showing that mental stimulation could protect against Alzheimer's disease and cognitive decline, they are more debatable [41]. There is evidence showing that cognitive stimulation can induce an increase in neuronal density [42].

#### 1.5.2. Food Supplements and Dementias

In recent years, many studies were aimed at confirming the preventive effect of food supplements, such as vitamins B6 and B12, folate, and vitamins E, C, and D [43].

Researches on vitamin B have achieved inconsistent findings. For example, a two-year treatment in 271 patients with vitamins B12 and B6 and homocysteine showed a significant difference in cerebral atrophy indexes, compared to placebo [44], while other reports indicate opposite results [45].

It has been shown that folic acid provides neuroprotective activity through an epigenetic mechanism, inhibiting the accumulation of the beta-amyloid peptide [46]. Conversely, a three-year treatment with vitamin E did not produce any protective effect, even when adding vitamin C to the treatment [47, 48]. Trials with vitamin D are also not definitive [49].

Finally, an absence of reduction in Alzheimer dementia and cognitive decline incidence after 6 years of *Ginkgo biloba* treatment has been demonstrated in high-quality trials [50–52].

## 2. Social Aspects of Dementia: From Caregivers to Related Costs

There are two main social aspects of dementia: The first point concerns the evidence that the Alzheimer's disease prevalence is likely to increase [1]. A second aspect pertains to people professionally involved in the assistance of the patients (caregivers) and the workload and costs that this assistance imposes to the family members [53]. For example, developed countries face increasing health expenses, both in absolute terms and as a percentage of the gross domestic product [54]. Since neurological and psychiatric diseases in Europe afflict more than 14% of the population, representing about 20% of health expenditure, this implies a segment of considerable public spending [55].

### 2.1. Caregiver Interventions and Family Engagement in Dementia

The term “caregiver” is used more in the United States, Canada, and China, while “carer” or “caretaker” is more common in the United Kingdom [56].

In any case, we can consider the following:

- Informal or primary caregivers, who play the role of caring for a family member
- Formal caregivers who carry out the role of assistance (doctor, nurse, social worker, etc.)

A management strategy is required to assess the cost/benefit ratio of treatments, and the expansion of the number of patients with dementia disease requires an acceleration of efforts in these fields. This explains why international research has developed considerably on this point. Chronic diseases have led to financial difficulties in public health systems. Hence, the role of informal caregivers is also important in evaluating economic and health analyses [57].

During preclinical dementia, when the disease appears and develops until its terminal phase, the patient poses complex health issues to social workers and family caregivers, who cannot always find the correct answer from the health and welfare services. These services, however, have not always reached a widespread and effective organizational model that can take into account the complexity of needs of the elderly [57].

Indirect costs account for about 75% of total cost of dementia, the most in the overall expenditure of the disease. They mainly consist of a loss of productivity, on individuals, families, and society. This very high impact is due to the loss of autonomy of patients and to the resistance of family members to take care of them, with consequences on health and welfare costs [58]. To the cost of diagnosis, the cost of home care and support for families must also be added: shortly after the first symptoms of the disease, a patient with Alzheimer dementia will need intensive assistance, even for 24 hours a day.

A percentage over 80% Alzheimer's patients is assisted at home, but during the evolution of the disease, two-thirds of the patients incur very serious behavioural problems that lead to stress issues for family caregivers, as well as causing an increase in the demand for professional care, with proportional greater economic commitment [57, 58].

Family members usually provide assistance to patients with dementia: professional support is required in the most serious cases. From the point of view of state balance, care provided by family members is considered to be free of charge. The assistance provided by the caregiver, on the other hand, is a real consumption of resources and a social cost. Consequently, a link can be established between the informal cost of assisting the patient and the social cost of the illness. In addition to the costs mentioned above, there are also intangible costs, i.e., the impact due to the physical and psychological suffering of the patient and relevant family members, which have social and human importance. Only towards the end of the last century, social policies and services have identified caring as a theme to pay attention to.

Patients living in caring structures usually have a lesser quality of life than those at home. Nevertheless, along the evolution of dementia, it may happen that the informal caregiver starts lacking the strength to assist the patient. Therefore, in this situation, a possible solution is the institutionalization by a private structure or the identification of a professional figure who will assist the patient at home [57, 58].

The advances in the technology of healthcare devices, including electronic health records, portal technologies, and wireless communications [59, 60], will probably have a key role in future dementia care. For example, tools for remote surveillance are very important and they give an important contribution to improving and making safer the environment in which patients and families live [59, 60].

There are also a few European experiences about neighbourhoods inhabited by patients and caregivers.

The city of Hogewey (Netherlands) [61] has been a European example of this since 1993, while in Rome, Italy, there is the Alzheimer's Village of the Fondazione Roma, in the Bufalotta district [62], where 100 patients are hosted free of charge in 14 houses, gathered around a square, and connected to everyday services, including a bar and a minimarket.

Also in the Lombardy region, there have been active interventions of this type, such as “Paese Ritrovato” (retrieved village) [63], launched a few months ago by the Cooperativa La Meridiana of Monza. It includes an area of 14,000 square meters, where 64 patients are housed, living in 8 apartments, with single rooms, assisted bathrooms, living areas, and a common kitchen; here, the hosted patients can keep external contacts and implement forms of sociality. The place is self-sufficient and, thanks to the presence of shops, allows patients to move in a protected yet autonomous way.

Another fundamental service for people living with dementia is represented by the role of Diagnostic Therapeutic Care Pathways (DTCP). The DTCP is an organization that covers both clinical and care aspects and is concerned in addressing them with the required harmonization [64].

They are the bodies that support the patient and his family in a straightforward way along the course of the disease, helping recognize and properly interface the different parts of the disease support system. The governance of the process should be shared, with different roles and responsibilities, between the general practitioner and the cognitive deterioration and dementia centres.

### 2.2. Costs Related to Dementia

The economic impact of dementia is a vast and continually growing figure, but it is still difficult to quantify [54]. The patients, their families, and carers suffer economically and in terms of quality of life [55]. Cost of dementia on the society is the sum of all expenses concerning goods and services used to prevent, diagnose, treat, and cope with the disease [65]. Per capita costs are divided into three subcategories: direct medical costs (e.g., drugs, medical and social services, hospital resources, and professional caregivers), direct social care costs (paid and professional home care and residential and nursing home care), and costs of informal (unpaid) care. Informal care is valued using an opportunity cost approach, valuing hours of informal care by the average wage for each country. In addition, indirect costs have to be taken into consideration such as the loss of income by the patient and possibly by family members or careers. Among the studies that have evaluated the costs of dementia [66, 67], some have focused on the relationship between costs and severity of the disease. Mean cost increases with the progress of the disease for patients in community dwellings, with variations across countries. Generally speaking, cost estimates have increased for all world regions, with the greatest relative increases occurring in African and Eastern Asia regions (largely driven by the upward revision of prevalence estimates for these regions) [68]. Distribution of costs between the three major subcategories (direct medical, social care, and informal care) has not changed substantially. As reported in 2010 [1], direct medical care costs are modest and account for roughly 20% of global dementia costs, while direct social sector costs and informal care costs each account for roughly 40%. As the country income level increases, the relative contribution of direct social care sector costs increases and the relative contribution of informal care costs decreases [69]. The relative contribution of informal care is greatest in African regions and lowest in North America, Western Europe, and several South American regions, while the reverse is true for social sector costs [69].

Other studies focused on the economic impact of drug therapies, concluding that cholinesterase inhibitors may be cost-effective in a short-term perspective [70]. The neuropsychiatric disorders and behavioural and psychological symptoms of dementia (BPSD) significantly contribute to the overall costs of dementia care. Interventions targeted at BPSD may help to reduce the staggering societal costs of this illness [71, 72]. Finally, the functional dependency grade of patients and comorbid medical conditions also affect the disease cost [73, 74]. With respect to direct medical care costs, they account for about 20% global dementia costs, with a further 80% divided among direct social sector and informal care costs. The upward revision of dementia prevalence in the African regions drove one of the greatest relative increases in costs compared to the previous estimates in 2010 and showed also a greater contribution of informal care to the costs [75].

In Italy, the direct costs of the assistance sum up to over 11 billion euros: 73% of the cost is charged to families, and the average annual cost per patient is 70,587 euros, including costs for the National Health Service [76].

Due to the expected increases in the number of people with dementia in the next future [77], the cost of the resources needed to support patients with dementia will significantly increase. To address these concerns, many countries are actively developing action plans for dementia at a national level. These plans are generally informed by a scientific approach to determine the type of care to be provided, where it is best to provide it, and the skill required of the personnel in charge of delivering it [78].

Nevertheless, many of the economic analyses reported above do not take into account the likely decline in age-specific dementia incidence that has been mentioned earlier [18, 22]: if this is true, then some of the reported costs could be considered overestimates.

### 2.3. Dementia as a Public Health Priority at the International Level

With respect to dementia awareness, there is an increasing involvement of international organizations, such as the WHO, the Organisation for Economic Cooperation and Development (OECD), and the EU.

During the 9th session of the Conference of the Member States on the “Convention on the Rights of Persons with Disabilities (CRPD)” [79], the United Nations (UN) dedicated a slot to dementia reaffirming the importance of the recognition and respect of the rights of all persons with mental and intellectual disabilities. Other initiatives have been deployed in recent years to promote and support collaboration with countries at international, regional, and national levels, in response to dementia.

The WHO hosted in March 2015 the first Ministerial Conference on Global Action Against Dementia, to discuss the global challenges posed by dementia, which brought together ministers, researchers, experts, clinicians, and nongovernmental organizations (NGOs) from around the world. The goal was to pay attention to the socioeconomic burden associated with dementias and how it could be reduced, raising it at the top of the global public health agenda. As a first result, there was the drafting of the “Global Action Plan (GPA) on the Public Health Response to Dementia 2017-2025,” which was later adopted by WHO member states at the 70th World Health Assembly in May 2017 [80].

The primary goal of the GPA is to improve the quality of life of people (patients and caregivers) living with dementia, to promote respect for their situation, and to reduce the negative impact on communities and states. To that effect, a series of actions were promoted in the following areas:

1. Improving awareness on dementia; increasing public knowledge, acceptance, and understanding of the illness; and adapting the societal environment to this disease. This is considered a key point to enable people with dementia to improve their social participation, give a contribution in the community, and maximize their autonomy. It is expected that 100% of countries will have one or more working public awareness campaigns on dementia and that 50% of countries will have one or more dementia-friendly initiatives, to foster a more inclusive dementia society by 2025
2. Reducing the risk of dementia, by increasing the capacity of health and social care professionals to provide appropriate interventions to the population and to educate about modifiable risk factors for dementia. Managing them proactively can reduce the risk of developing dementia, and its progression can be delayed
3. Ensuring diagnosis, treatment, and care to patients, granting that the needs and preferences of people with dementia can be met and their autonomy respected. This result can be achieved through integrated, appropriate, community-based health, psychosocial, and long-term care and support focused on the patient and, where appropriate, on families and carers. It is expected that in at least half of countries, 50% of the estimated number of people with dementia will be diagnosed by 2025
4. Supporting family members and caregivers so that the implementation of solutions to deliver multisectoral care, support, and services for caregivers will help to meet their needs and will prevent a decline in their physical and mental health and social well-being. It is expected that, by 2025, 75% of countries will provide support and training programmes for caregivers and families of patients with dementia
5. Working on information systems for dementias, in order to start a systematic monitoring and evaluation process of the usage of health and social care systems. This action can provide the best evidence for policy development and service delivery and can improve the prevention, the accessibility, and the coordination of care for patients with dementia, from risk reduction to the end of life. It is expected that, by 2025, half of countries routinely will collect a core set of dementia indicators, through their national health and social information systems, with a period of two years
6. Promoting research and technological innovation, in order to increase the probability of effective progress towards better prevention, diagnosis, treatment, and care for patients with dementia. It is expected that, between 2017 and 2025, the output of global research on dementia will double

As a consequence of the adoption of the plan, the WHO launched a “Global Dementia Observatory,” oriented to the description and worldwide monitoring of the characteristics of dementia and the relevant public health responses, provided by different countries. For the setup of this observatory, Italy was invited to collaborate as a pilot country in order to test the feasibility of data collection and provide suggestions for the selection of key parameters and indicators.

### 2.4. Dementia as a Public Health Priority at the European Union (EU) Level

In recent years, the “Joint Action on Dementia (JA)” and the “Joint Programming Research Initiative” were the main tools that the EU used to leverage and translate into action the official documents that have been produced by the commission and the council in the field of dementias. The first of those initiatives, in which Italy has actively participated, is called “ALCOVE” and sees the majority of EU member states involved in research activities, using a common scientific approach and sharing experiences.

ALCOVE, which stands for Alzheimer Cooperative Valuation in Europe, ended in 2013: in this project, Italy coordinated the epidemiological section [81].

The participation of Italy in the second JA, which started in 2016 and is expected to last 3 years, has the main objective of translating the recommendations of ALCOVE into working implementations. The goal of the Act on Dementia Joint Action is to promote collaborative actions among member states, to increase the quality level of the lives of patients and families living with dementia. It will provide a practical pattern for policymakers developing and implementing their national dementia plans and strategies. The JA is aimed at providing practical and cost-effective examples of the core elements of good dementia diagnosis, care, and support [82].

In addition, Alzheimer Europe, the organization that brings together European associations concerned with the disease, recently presented the assessment called “European Dementia Monitor” [83], which covered almost all member states of the EU (with the exception of Estonia) plus non-EU member countries like Albania, Bosnia and Herzegovina, Jersey, Monaco, Norway, Switzerland, Turkey, and Israel, which even if outside the EU territory are officially associated with the EU. The analysis was developed on the basis of ten different categories, which included the availability and accessibility of care services, the reimbursement of medicines, and the deployment of initiatives in favour of patients living with dementia. The investigation showed significant differences between the 36 participating countries with no country scoring full points in all ten categories. Finland had the best ranking with an overall score of 75.2%, followed by England (72.4%), the Netherlands (71.2%), Germany (69.4%), Scotland (68.8%), and Italy (52.9%).

Finland had the highest score in terms of availability and accessibility of care services and, together with the Netherlands and England, also scored best on initiatives on inclusion and dementia-friendly community. Belgium, England, Ireland, Scotland, and Sweden ranked first in terms of reimbursement policies of drugs.

In these countries, in fact, all antidementia treatments are fully reimbursed by the health service, and there is in force a limit against the inappropriate use of antipsychotics. France, Germany, and Spain scored highest in the category of clinical trials, while Ireland and Norway were the first to recognize dementia as a national research and policy priority.

England, France, Germany, Israel, the Netherlands, Scotland, and Slovenia were distinguished by following Alzheimer Europe's recommendations on respect for the legal rights of patients with dementia and their families; Ireland ranked first in terms of recognized care and work rights, while Finland and Norway already ratified international and European human rights conventions.

Although Italy is the most committed and active country in European research collaborations, it only ranks halfway through the general ranking because of a lack of availability of and accessibility to care services, in addition to a low recognition of dementia as a public health priority [83].

#### 2.4.1. National Dementia Plan of Italy

In October 2014, Italy (one of the countries with the oldest people, as reported above) approved the National Dementia Plan (NDP) that was later presented at an international conference as part of the initiatives of the Italian Presidency of the EU; it is recognized that the plan contributed to strengthening the quality of cooperation within the European Commission [84].

The plan was approved as an agreement between the Unified Conference with local administrations, the government, the regions, and the autonomous provinces redacting the document “National Dementia Plan - Strategies for the Promotion and Improvement of the Quality and Appropriateness of Welfare Interventions in the Dementia Sector.”

This plan was aimed at promoting and improving interventions on specialist therapeutic aspects of dementia and at supporting patients and their families throughout the care process. Furthermore, it provided strategic indications for the promotion and improvement of new strategies “starting from the assumption that, as in all chronic degenerative pathologies in which the pharmacological approach is not decisive in modifying its natural history, it is necessary to provide an articulated and organic set of care pathways, according to a philosophy of integrated management of the disease.” Since its launch, the NDP has represented the most important national public health goal that aligns Italy with the dementia policies carried out by other Western countries. The awareness of the complexity of the dementia phenomenon, with all its implications on the social fabric, requires an extraordinary commitment by the central and regional institutions in close collaboration with the associations of family members and patients. This commitment has to be directed towards the development of governance ability for complex phenomena, an indispensable tool for facing this social and health emergency [85].

It has been established that the implementation of the plan has to be monitored through the “Monitoring Table of the Implementation of the National Plan for Dementia (NDP),” coordinated by the Ministry of Health, which is aimed at reverting into concrete actions the objectives of the plan itself.

The main objectives of the document are as follows:

Objective 1. The identification of interventions and measures in the field of health and social-health policy, with the aim of increasing the awareness of the general population, of people with dementias and their families and professionals, each for its own level of competence and involvement; prevention, early diagnosis, treatment, and care of people with dementia, also with attention to early-onset forms; achieving, through research support, improvement in caring for and improving the quality of life of patients with dementia and their caregivers. A further goal is to organize and implement an epidemiological survey, focused on planning and improving care, for effective and efficient management of the disease
Objective 2. The creation of an integrated network on dementias and the deployment of integrated management actions to promote prevention, early diagnosis, and appropriate intersectional policies, also with a view to reducing discrimination. A further objective is to converge to a common and homogeneous assistance process, paying particular attention to social inequalities and to conditions of fragility and social and health vulnerability
Objective 3. The implementation of strategies and interventions to increase the quality of caring, improving the ability of the NHS to deliver and monitor effective services, through actions such as rationalization of the supply and the use of working methods based primarily on the appropriateness of the provided services. Furthermore, promoting strategies to improve the quality of care to patients with dementia at home, in residential and semiresidential facilities, and during the entire evolution of illness; eventually developing the appropriateness of the use of drugs, technologies, and psychosocial interventions
Objective 4. The increased awareness and reduction of stigma, for the improved quality of life of patients living with dementia and their families, through correct information on the disease and the available services; the objective here is to facilitate the access to them as early as possible
Objective 5. The improvement of the quality of life and care and the promotion of a full social integration for patients with dementias, also through strategies of personal and family involvement
Objective 6. The promotion of any form of participation, in particular through the involvement of families and associations

After its emanation, the plan implementation was monitored at the regional and national levels by evaluating the activities of permanent confrontation and monitoring as reported in Objective 1.

Besides the monitoring activity, the future objectives reported in the NDP are aimed at the following:

- Identification of quality standards and measures for monitoring plan activities
- Elaboration of guidelines concerning crucial aspects linked to the disease such as diagnosis communication, informed consent, and use of legal options
- Discussion on ethical issues such as advance directives and accessibility to palliative care
- Formulation of guidelines dedicated to patients developing dementia in working age and focused on early-onset dementias [86]

For the achievement of these goals, the NDP required the activation of a permanent steering group on dementias in order to monitor the transposition and implementation of the NDP.

After the approval of the NDP, many activities were carried out to monitor the state of transposition and implementation of the NDP at the regional level with the establishment of two working groups to formulate the technical documents “Diagnostic Therapeutic Care Pathways” and “System and Information Flows,” and two national guidelines have been drawn up to better achieve the objectives of the NDP [85].

As reported in the Ministry of Health's document “General Directive for Administrative Activity and Management,” approved in March 2019 [87], the monitoring of the NDP is being carried out at the central level together with the 21 Italian regions and, in particular, after the approval of the two above-reported technical documents during the Regional Unified Conference in 2017; the formulation of a further document on ethical issues will be completed.

Furthermore, the mental health working group created in January 2019 by the Minister of Health will proceed to

- verify the implementation of the scientific guidelines, including the agreements established in the State-Regions Conference and Unified Conference as a commitment of the National Action Plan for Mental Health
- verify the appropriateness of the treatment and rehabilitation courses provided by the territorial health services and by the psychiatric diagnosis and treatment services
- explore the existence of any eventual critical issues in the territorial health services and elaborate proposals for their solution and for optimizing the network of services, through their strengthening
- propose operational and regulatory actions to facilitate the implementation of the most appropriate models of intervention for the psychosocial diagnosis, treatment, and rehabilitation of people with mental illness, aimed at reducing compulsory and voluntary mental health treatments and mechanical and pharmacological restraints [87]

Nevertheless, several relevant issues still negatively affect the management of the phenomenon of dementia in Italy. In particular, there are many disparities of resources and services across the different regions which demonstrate the lack of national standards. The different aspects of dementia management (i.e., diagnosis, assistance, and rehabilitation) are still addressed in separate moments and processes in different parts of the nation, severely limiting the possibility of actually implementing an integrated approach to dementia [86].

In addition, so far the National Dementia Plan has not been financially supported and there are numerous activities aimed at raising the awareness of the competent authorities to identify funds for the implementation of the projects and services established by the plan.

In conclusion, even in the presence of several unsolved situations, it can be said that to date, Italy is improving the management of the dementia problem both nationally and in Europe, to develop and optimize new health and social strategies for improving the quality of life of the people affected by dementia and for greater appropriateness of care interventions.

## 3. Conclusions

We aimed to review the most important social aspects of dementia, briefly reminding the current epidemiology of the disease, with a special attention to the situation in developing countries and sub-Saharan Africa. We concisely listed the modifiable risk factors for dementia and the pharmacological and nonpharmacological treatments including lifestyle strategies. Then, we focused on the caregivers' interventions and the costs related to dementia.

They can be useful to allow policymakers to define their choices according to the economic and social situations of their countries [53]. This is of particular importance for developing countries, where dementia prevalence is expected to increase in the next decades, so that programs focused on pharmacological but above all nonpharmacological treatments (i.e., lifestyles) should be taken as soon as possible. Conversely, in the US and Europe, where life expectancy is already high, different strategies should be considered, taking into account the cost of these interdisciplinary efforts including, as previously reported [53], physicians, neuropsychologists, and economists.
